# Dual-Beam THz Spectrometer with Low-Aberration Optics and Off-Axis Multipixel Photoconductive Emitters for Reduced Systematic Errors

**DOI:** 10.1021/acsphotonics.4c01934

**Published:** 2025-01-11

**Authors:** Nishtha Chopra, James Lloyd-Hughes

**Affiliations:** Department of Physics, University of Warwick, Coventry, West Midlands CV4 7AL, U.K.

**Keywords:** THz spectroscopy, dual beam, systematic, reproducibility, low aberration, transmission

## Abstract

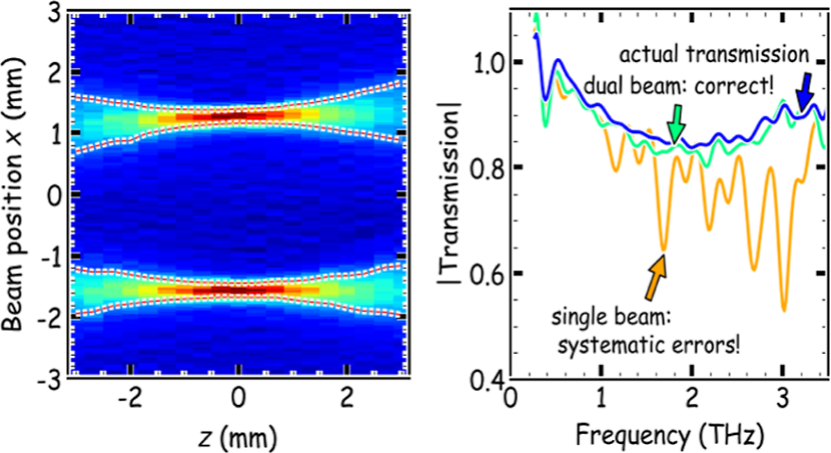

A dual-beam THz spectrometer is reported that substantially reduces the influence of systematic errors in THz time-domain spectroscopy such as those caused by variations in femtosecond laser power or the environmental temperature and humidity. Dual THz beams with single-cycle waveforms were generated simultaneously using a dual-pixel interdigitated photoconductive antenna, allowing the simultaneous acquisition of sample and reference data in the spectrometer using the same optical components. A low-aberration optical geometry ensured diffraction-limited spatial profiles for both beams despite their off-axis propagation and was validated experimentally by measuring frequency-dependent beam profiles and theoretically via physical optics calculations. Although the experimental amplitudes and absolute phase spectra of both beams were very similar, we further provided a correction procedure to eliminate these small differences. The robustness of the dual-beam spectrometer design was evaluated by measuring the complex transmission of a thin plastic sheet after intentionally introducing a change in the relative humidity of the THz beam path. The dual-beam THz spectrometer was effective at removing systematic errors in the amplitude and phase by simultaneously measuring the two THz beams under the same conditions.

## Introduction

Terahertz time-domain spectroscopy (THz-TDS) is a phase-sensitive detection technique crucial for analyzing the material properties of semiconductors, superconductors, and biomaterials.^1,2^ Ensuring reproducible and stable measurement conditions remains challenging due to the complexity of THz spectrometers, which rely heavily on the synchronous operation of their subsystems. Sources of error can be regarded as introducing random noise, such as shot-to-shot fluctuations caused by the inherent noise of a femtosecond laser, or a systematic error, such as a change in optical alignment during measurements. Errors directly impact the efficacy of the parameter extraction process:^3^ random noise controls the precision of results, while systematic errors influence the accuracy of material properties derived from the experiment. Some standard techniques work effectively to remove random noise: modulating the THz signal at high frequency and reading it via a lock-in amplifier to remove Johnson noise^4^ or increasing the number of measurements per second to improve the signal-to-noise ratio achievable in a given time.^5^ However, fewer studies have tackled the systematic errors in THz-TDS.

The conventional method for material characterization typically involves taking sequential measurements of the time-varying electric field using one THz beam: first, a reference measurement is taken, which is then followed by a measurement with the sample present. Ideally, the sample and reference measurements are taken back to back as quickly as possible, to lower the chance of systematic errors associated with slow drifts in the spectrometer’s performance, for instance, created by temperature or humidity changes. Double-modulation terahertz spectroscopy achieves this by rapidly translating the sample into and out of the THz beam,^6,7^ resulting in a differential signal sensitive to subtle variations in absorption and phase. While the sample is inserted and removed relatively fast (at a few Hz), overall data acquisition is relatively slow, as the modulation signal has to be acquired by averaging over a few repeats of the sample modulation frequency, for each time delay in the THz waveform (step scan mode). This technique is thus not compatible with modern fast-scan THz data acquisition methods that acquire THz waveforms at rates of faster than a few Hz (e.g., rapid delay lines; asynchronous optical sampling). For small samples, the sample and reference are also located only at the correct positions for a fraction of the experiment, making for a relatively low-duty cycle of time spent usefully acquiring data.

An alternative approach to reduce “drift”-like systematic errors is to perform self-referencing, where the time-domain waveforms contain a component that has not interacted with the material of interest but which is recorded at the same experimental time. Such a self-reference can be provided by an internal reflection of a fraction of the THz beam within the sample or from a support substrate.^8^ However, such methods rely on accurate knowledge of the substrate and make assumptions about sample geometry such as the absence of air gaps, which can introduce systematic errors during data postprocessing. THz ellipsometry^9^ provides another method to avoid drift, where comparing the s- and p-polarized reflected (or transmitted) spectra for s- or p-polarized input can determine the complex refractive index of materials without a reference or a complex calibration procedure. In generalized THz ellipsometry,^10^ two photoconductive antennas were used to generate two orthogonally polarized THz beams, which were then combined with a THz polarizer, reflected from a sample, split into orthogonal polarizations, and detected by two photoconductive detectors. While an important step forward, compared to a standard THz time-domain spectrometer, the system had roughly double the components,^10^ adding to the system’s cost and complexity. Further, the different THz beams did not travel the same path length (e.g., p-input and s-input channels were separated by 25 ps), making the system susceptible to changes in water vapor absorption if humidity levels changed.

At visible wavelengths, the challenge of drift between sequential measurements has been solved using double-beam spectrometers, wherein one beam interacts with a sample, while the other acts as a reference. Dual-beam spectrophotometers are readily available from a number of commercial suppliers. Light from one source is split into two beams by a beamsplitter: both beams are detected simultaneously (or by rapidly alternating between each beam) to help eliminate errors introduced by slow changes, such as drift in the intensity of the light source. Dual-beam spectroscopy has not been widely adopted or explored in the THz range by the academic and industrial users of THz methods, although it has been proposed in patent applications^11,12^ by using two independent THz time-domain spectrometers driven by the same fs laser (i.e., two separate THz sources, two independent sets of optics, and two THz detectors).

In this article, we introduce a convenient dual-beam THz spectrometer featuring a compact THz source that produced two THz beams, which propagated through the same optical components and were measured simultaneously by a single detector capable of detecting the spatially separated THz beams. By using two THz beams propagating along the same optical path, this approach mitigates against long-term drifts that cause systematic errors while reducing the system complexity and cost. After describing the experimental setup for dual-beam THz spectroscopy, we validate the spectrometer’s optical design using physical optics simulations. In the Experimental Results, we first demonstrate that the two THz beams had similar spatial profiles but were well-separated by the design distance in an intermediate focal plane. To allow for differences in path length of the two probe beams, we report how a Fourier time-shifting algorithm can synchronize the measurement time window for both beams. We then define a dual-beam transmission formula for spectroscopy that allows systematic errors to be removed and which corrects for any amplitude and phase differences between the two beams. Finally, we created systematic errors in a controlled manner to directly compare the relative performance of single- and dual-beam THz spectroscopy when measuring a thin plastic film. While the traditional single-beam approach was very sensitive to variations in atmospheric humidity levels, our dual-beam method reliably reproduced the correct complex transmission.

## Dual-Beam Spectrometer Design

**Figure 1:**
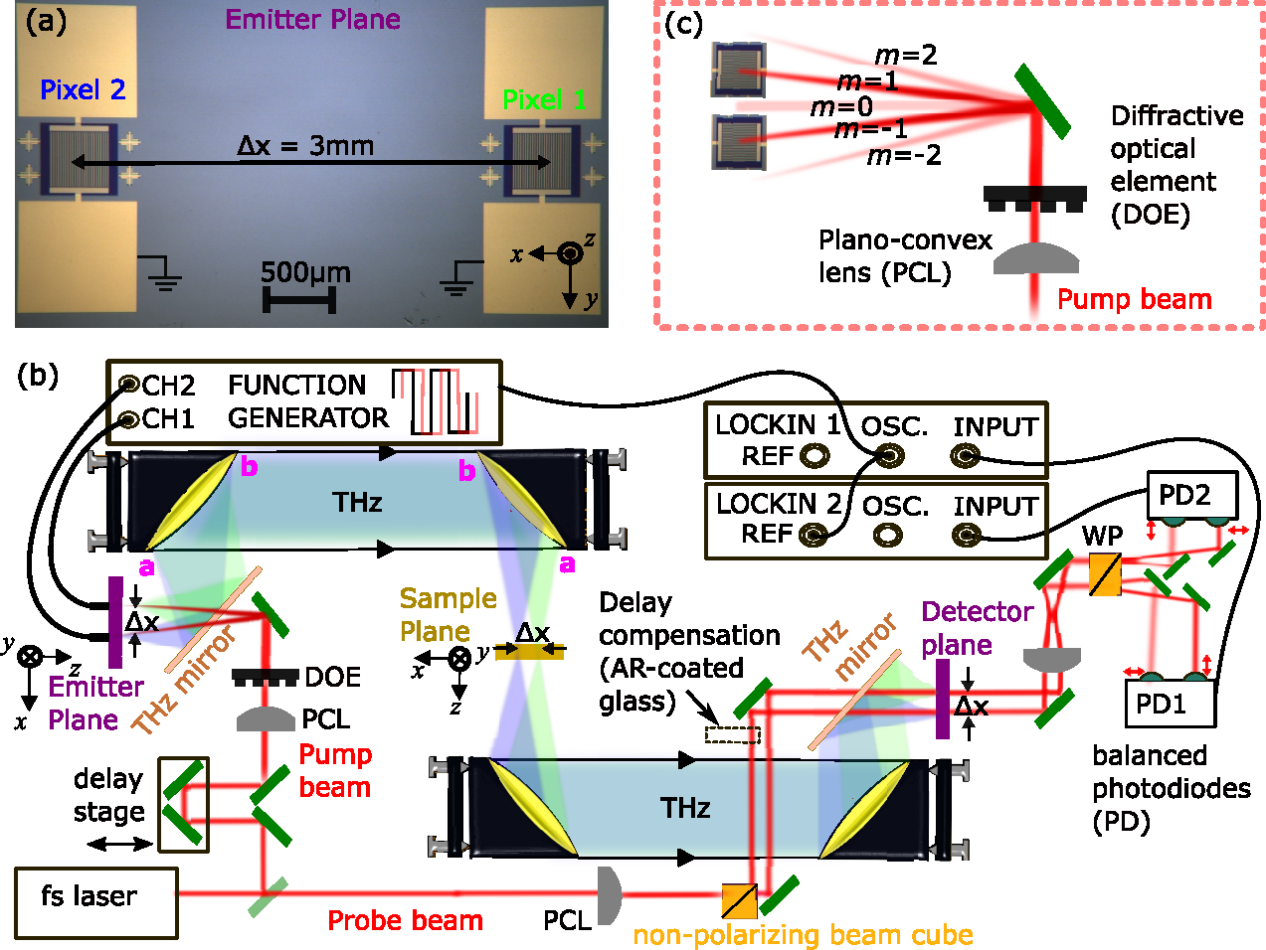
(a) Microscope image of dual-pixel THz emitters placed in the emitter plane, with pixel centers spaced by Δ*x* = 3 mm. (b) Dual-beam THz spectrometer showing THz beams 1 (blue shaded area) and THz beam 2 (green area). The off-axis parabolic mirrors (OAPMs) are in an (*a*, *a*)(*a*, *a*) geometry,^17^ such that a marginal ray hitting side *a* (pink) remains on side *a* on each subsequent OAPM. Inserting different thicknesses of antireflection (AR)-coated glass (dashed box) can compensate for different arrival times of the two THz beams. Red arrows near the balanced photodiode pairs indicate horizontally and vertically polarized probe beams, created after the Wollaston prism (WP). (c) Detail of how the diffractive optical element (DOE) and plano-convex lens (PCL) create two pump beams primarily in diffraction orders *m* = ± 1, with low intensity in the other orders.

Multipixel photoconductive emitters have been explored for polarization modulation,^13,14^ generating circularly polarized^5^ or cylindrical vector beams,^15^ and THz beam shaping.^16^ However, in all cases, the emphasis was on producing a single THz beam that was close to the optical axis of an optical system. In order to create a compact THz spectrometer with two independently controllable and spatially separated THz beams, we adopted an approach where the THz sources were placed close to, but away from, the optical axis. The dual-beam THz time-domain spectrometer is illustrated in Figure 1. Two independent photoconductive emitter pixels, each consisting of interdigitated electrodes, were fabricated on the same SI-GaAs substrate, following the photolithography processes outlined previously.^16^ The distance between the center of each pixel was Δ*x* = 3 mm, as shown in Figure 1a, and each pixel had an active area of 500 × 500 μm. The pixels were positioned with an offset of *x* = ± 1.5 mm from the optical axis: this separation was chosen to provide a convenient separation between the sample and reference sampling areas in the spectrometer’s sample plane. Changing the photolithography process or cleaving and separating devices by a custom spacing would readily allow alternative separations between pixels to be achieved. Devices were mounted onto a copper-strip printed circuit board with bias and ground pins soldered individually for each pixel.

The experimental implementation employed a Ti:sapphire mode-locked laser (Spectra Physics Mai Tai) oscillator, featuring a 100 fs pulse duration and an 80 MHz repetition rate, operating at a central wavelength of 800 nm. The laser beam was split into pump and probe beams to photoexcite the emitter and detect THz radiation with a single 200 μm-thick GaP detection crystal. The pump beam was further divided in two using a commercial transmissive DOE, designed to efficiently produce light primarily in the *m* = ± 1 diffraction orders with a full separation angle of θ = 1.91° and a 100 mm focal-length PCL. This enabled the excitation of the individual pixels using two beams (300 mW per beam) that became spaced by 3 mm at the emitters (Figure 1c). The THz radiation emitted backward (reflection geometry) was collected and used, to avoid dispersion and absorption in the GaAs substrate at higher frequencies.^18^ The use of a standard silicon lens with the dual emitters was avoided, as spherical lenses only work well for on-axis point sources.^19^ The conductive side of a fluorine-doped tin oxide (FTO) THz mirror reflected the THz radiation into an imaging system (Figure 1b) consisting of two pairs of OAPMs with 76.2 mm focal length and 50.8 mm diameter and aligned in an (*a*, *a*)(*a*, *a*) configuration.^17^ These mirrors guide the two beams in free space, ensuring a diffraction-limited spot size at both the sample and the detector planes. The system was designed to have minimal wavefront distortion and aberration, by staying within the tolerance limits for beams originating from off-axis points in the emitter plane.^17^

On the detection side, the probe beam was split 50:50 using a nonpolarizing beam cube. The two probe beams were used to perform electro-optic sampling of the two THz beams, which were focused onto a GaP crystal in the detector plane by the last OAPM. A second FTO mirror was used to combine the two THz beams and two IR probe beams collinearly. A mechanical delay line varied the arrival time of the pump beam, changing the arrival time of both THz pulses relative to that of the fixed probes. In this implementation, beam 2 had a slightly longer path length given by Δ*x* = 3 mm, which was compensated by introducing an AR-coated glass window to the optical path length of beam 1, as seen in Figure 1b.

Using a dual-channel function generator, the two photoconductive emitters were amplitude modulated with independent square wave voltages at the same frequency 100 kHz and amplitude *V*_pp_ = 20 V. The voltage applied to emitter 2 was phase-shifted by 90° relative to emitter 1, to prevent optical cross-talk at the detection side (so that the phase-sensitive electro-optic sampling of THz beam 1 did not detect the tail end of THz beam 2’s spatial profile, see Supporting Information). Utilizing a Wollaston prism, the orthogonal components of both beams were created and then sent to two pairs of balanced photodiodes. Subsequently, two lock-in amplifiers individually registered the electro-optic signal of each THz beam.

**Figure 2:**
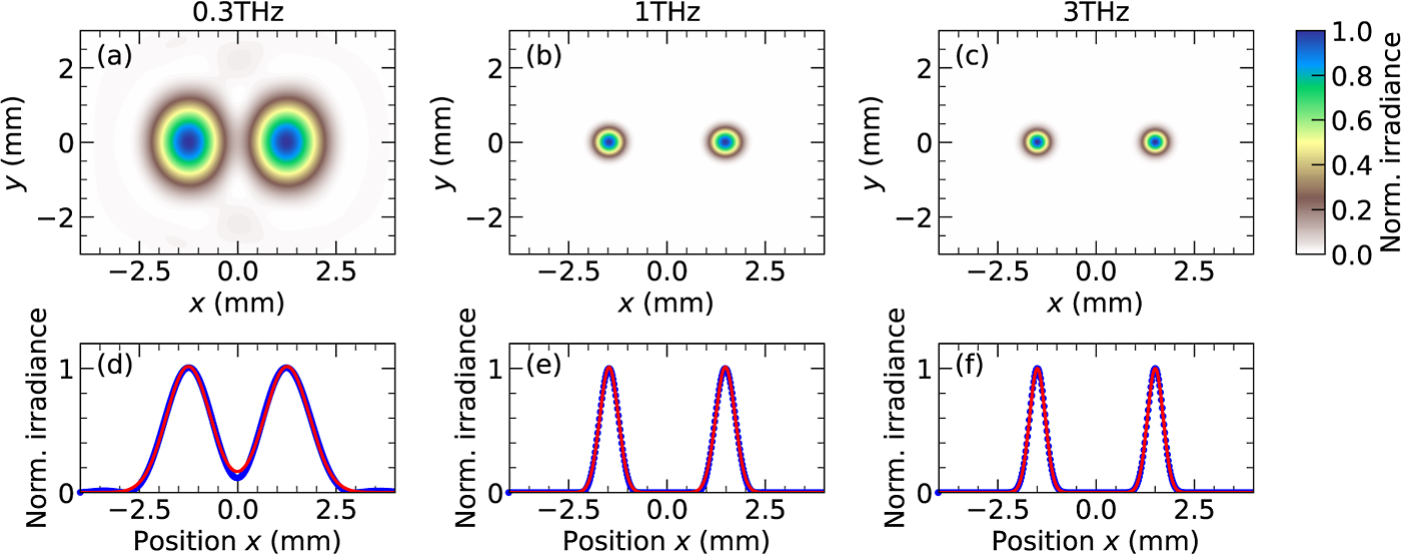
Simulated beam profiles for the dual-beam THz spectrometer at the sample focal plane. (a) At *f* = 0.3 THz, the beams have a small overlap with a nonzero intensity near the origin (0,0). Beam profile for (b) *f* = 1 THz and (c) *f* = 3 THz. (d–f) Corresponding cross-sectional beam profiles along *x* at *y* = 0 mm from simulation (blue points) and fits using Gaussian profiles (red lines) with standard deviations σ = 0.56 mm, σ = 0.24 mm, and σ = 0.20 mm for 0.3 THz, 1.0 THz, and 3.0 THz.

To validate the broadband design of the THz optical system theoretically, we used the physical optics module in the optics simulation software Ansys Zemax to propagate the wavefront of both THz beams throughout the optical setup. This approach uses a Fresnel diffraction algorithm to propagate a coherent beam through an optical system as it diffracts along the propagation direction, *z*, and is collimated and focused. The initial THz beams each had a Gaussian profile with waists *w*_0_ = 0.4 mm and centered at *x* = ± 1.5 mm in the object plane (emitter plane). The divergence angle θ of each beam was determined by Gaussian beam theory according to tan θ = λ/(π*w*_0_) for wavelength λ. The numerical grid was dynamically resampled at different positions in order to accurately model the size and shape of the beams. At all frequencies, the two beams are circular, and beam cross sections are close to Gaussian in shape, evident from fits to the *x* cross sections shown in Figure 2d–f of the form . The beams are well-separated by the designed spacing of 3 mm as a result of the 1:1 magnification of the optics (equal focal length OAPMs). The excellent beam quality even for off-axis beam points is a feature of the (*a*,*a*) orientation adopted for each OAPM pair,^17^ which has minimal aberrations. Here, (*a*,*a*) refers to the relative orientation of each OAPM pair such that marginal rays always hit the same side of the OAPM (see pink “a” labels in Figure 1b). At 0.3 THz, in Figure 2a,d, the THz beams are larger than at higher frequency: the diffraction-limited spot size is bigger for longer wavelengths (σ = 0.56 mm at 0.3 THz) than at shorter wavelengths (σ = 0.24 mm at 1.0 THz). The intensity does not completely drop to zero between the beams on the optical axis (*x* = 0, *y* = 0) for 0.3 THz, suggesting that at lower frequencies, there may be some spatial overlap of the two beams. At higher frequencies (1 THz and 3 THz frequencies), the two beams are more clearly distinct, with a higher peak intensity and smaller spot size (Figure 2b,c). Therefore, the dual-beam approach can be used to perform spectroscopy at frequencies of 0.3 THz and higher, such that a sample placed in one beam (covering *x* ≥ 0) does not attenuate too much of the reference beam (*x* < 0).

The second pair of OAPMs propagates the two THz beams from the sample plane to the detector plane (Figure 1), where diffraction-limited foci with similar optical performance are expected,^17^ including some overlap of the two beams at lower frequencies. To prohibit cross-talk in the detection plane in the experiment (e.g., probe beam 2 sampling the edge of THz beam 1), the two beams can be modulated at different frequencies or with orthogonal phases, such that no cross-talk is observed between the beams (Supporting Information).

## Experimental Results

### Dual-Beam Spatial Profiles

**Figure 3:**
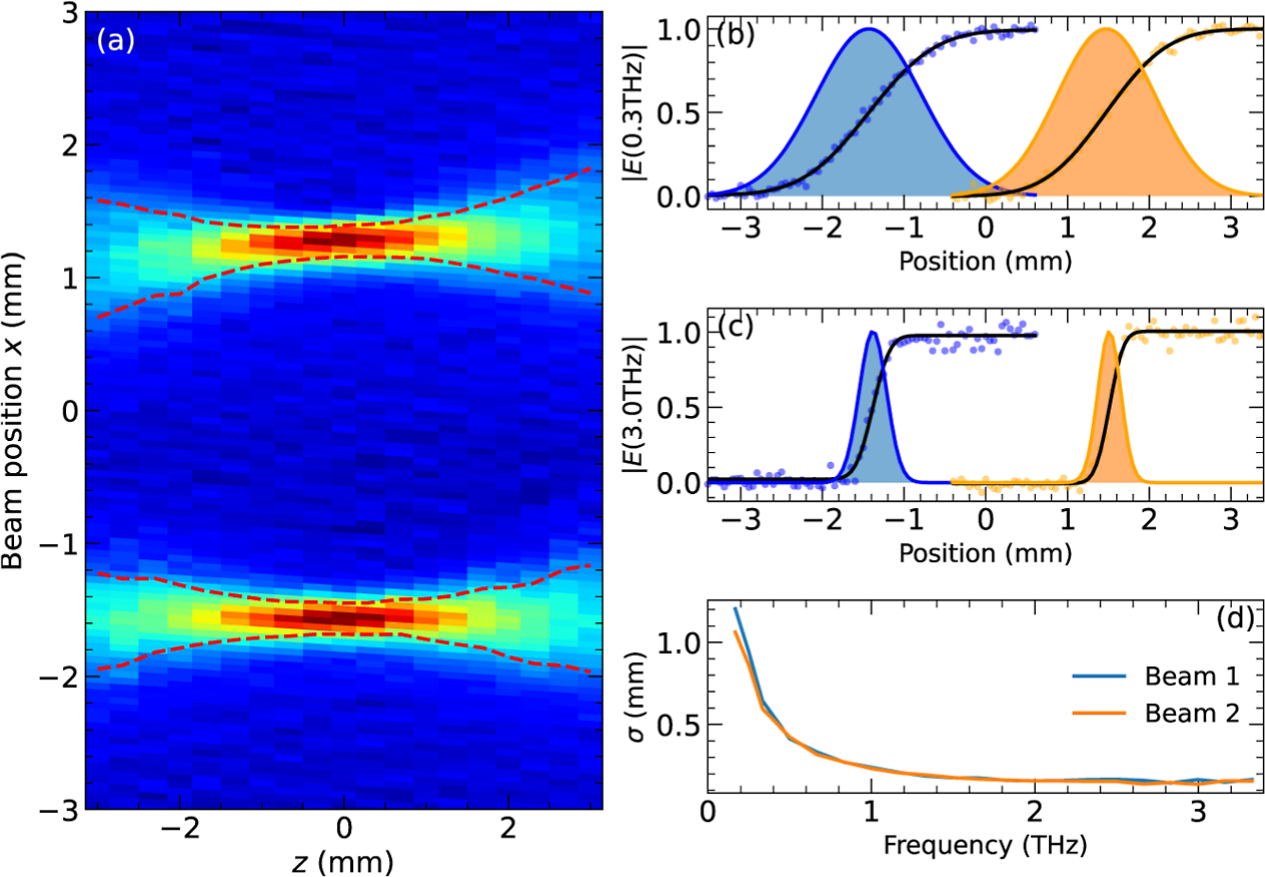
(a) Profiles of the dual THz beams, from knife-edge scans along *x* at different distances *z* along the propagation direction, at the time-domain peaks of beam 1 (*x* < 0) and beam 2 (*x* > 0). Dark red corresponds to the highest irradiance, while dashed lines show the beam waists at ± σ from the beam centers. (b) Frequency-resolved spatial profiles along *x* in the sample plane (*z* = 0) at *f* = 300 GHz. The experiment measured the total electric field passed from *x*′ = −∞ to *x*′ = *x*, |*E*_T_(*x*,*f*)| = |∫_–∞_^*x*^*E*(*x*^′^,*f*) d*x*^′^|, for beams 1 (blue points) and 2 (orange points). Black lines indicate fits using an error function to model the data, while the shaded areas show the corresponding electric field Gaussian profiles, |*E*(*x*, *f*)| = ∂|*E*_*T*_(*x*,*f*)|/∂*x*. (c) as (b) but at 3.0 THz. (d) σ from Gaussian fits versus frequency. Identical beam profiles were obtained for both beams.

To characterize the spatial performance of the spectrometer, we used the knife-edge method to characterize both beams, principally to check if they were spatially separated. We recorded the electro-optic signal at the time-domain peaks of each THz pulse as a knife edge was moved into the beam along the *x* axis, in 50 μm steps. The beam profile was obtained by numerical differentiation along *x*, and this process was repeated at different *z* positions to track the beams’ evolution along the propagation axis. As shown in Figure 3a, the two beams were tightly focused in the sample focal plane (*z* = 0) and were separated by a distance Δ*x* = 2.8 mm, close to the device’s designed separation of 3 mm. This demonstrates that the dual-beam spectrometer approach produces well-separated THz beams, suitable for sample and reference measurements.

Gaussian beam fits of the form  were performed at each *z* for both beams, *n* = 1 and 2. The dashed lines in Figure 3a show the positions μ_*n*_(*z*) ± σ_*n*_(*z*), corresponding to the electric field dropping by a factor of *e*^–1/2^ or the intensity dropping to 1/*e*. The values and *z* dependence of the 1/*e* widths can be seen to be similar for both beams. The beams were tightly focused within 1 mm of the focus, where they had the highest amplitude (dark-red areas).

Using frequency-dependent knife-edge measurements,^16^ we then investigated the wavelength dependence of the beam profiles at the focus (*z* = 0). We recorded time-domain waveforms at different knife-edge positions (steps of 50 μm along *x*) and Fourier transformed to get the amplitude spectra of the electro-optic signal (Figure S2). As reported in Figure 3b, the beams had similar amplitude profiles at *f* = 0.3 THz (points), which were consistent with error function fits (black lines) corresponding to Gaussian beams with σ_1_ = 0.60 mm and σ_2_ = 0.64 mm (shaded areas). The beam profiles were sharper at higher frequencies, for instance, with σ_1_ = σ_2_ = 0.15 mm at *f* = 3.0 THz, evident in Figure 3c. The similarity between the profiles can be further seen in Figure 3d, where it is evident that σ_1_ ≃ σ_2_, and both beams had similar frequency-dependent sizes. The frequency dependence of the spot size can be understood with reference to Gaussian beam theory and the wavelength dependence of the beam divergence (see Supporting Information): σ is frequency-independent at higher frequencies, with σ_*n*_(*f*) ≃ 0.15 mm for *f* ≥ 1.5 THz, while σ_*n*_ ∝ λ ∝ 1/*f* at longer wavelengths (*f* < 1 THz) where the THz beam starts to overfill the first OAPM.

The low-aberration design of the spectrometer allowed good spatial separation of the two beams for frequencies of 0.3 THz and higher, allowing dual-beam spectroscopy measurements to be performed by placing a sample in one of the two beams. The radius of a Gaussian beam remains roughly constant within the Rayleigh range, a distance of *z*_*R*_ = 2πσ^2^/λ from the focus. This illustrates the level of accuracy required when positioning a sample at the focus along *z*. For example, using σ = 0.64 mm at 0.3 THz (Figure 3d), the Rayleigh range is *z*_R_ = 0.77 mm, or at 3 THz with σ = 0.15 mm, it increased to *z*_R_ = 1.4 mm.

### Time and Frequency Domains

**Figure 4:**
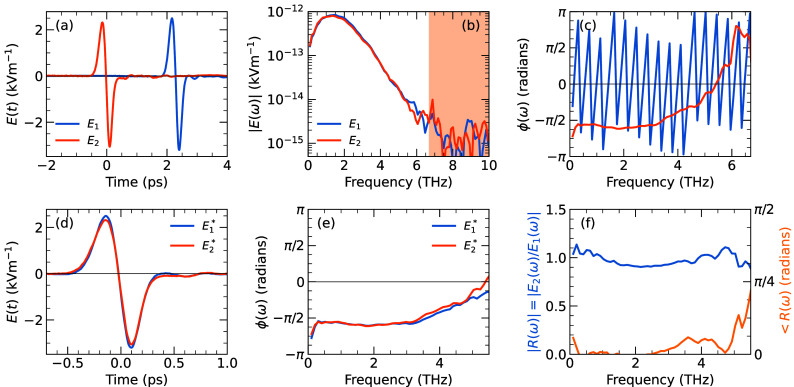
(a) Time-domain THz electric fields of beams 1 (blue) and 2 (orange) with a nitrogen-purged beam path. (b) Amplitude spectra of beams 1 and 2. Spectra are very similar, with a bandwidth of 6.5 THz before reaching the noise floor (shaded area). (c) Absolute phase spectra (without unwrapping) of beam 1 and 2. The rapid change in phase in beam 1 is a result of the extra 2.3 ps time delay. (d) Time-domain waveforms *E*_1_^*^(*t*) and *E*_2_^*^(*t*), obtained by the inverse transform of time-shifted spectra. (e) Phase spectra of time-shifted data, *E*_1_^*^(ω) and *E*_2_^*^(ω), are very similar for beams 1 and 2. Chirp in both pulses (a nonlinear increase in the phase with frequency) is evident at higher frequencies, as discussed in the text. (f) Amplitude and phase spectra of the ratio *R*(ω) = *E*_2,b_^*^/*E*_1,b_^*^. The similarity of the two beams is evidenced by the ratio being close to 1 and having low phase difference.

The THz electric field pulses *E*(*t*) produced by each emitter are shown in Figure 4a, where 10 scans each taking 10 s acquisition time were averaged. Single-cycle THz pulses were obtained for both THz beams 1 and 2, with pulses in beam 1 arriving 2.3 ps later than beam 2, as a result of the differing optical path lengths of probe beams 1 and 2. A phase- and amplitude-accurate discrete Fourier transform routine (Supporting Information and ref^20^) was then used to calculate the complex frequency spectrum of each beam, . Amplitude spectra in Figure 4b show good spectral coverage from 100 GHz to 6.5 THz, with very similar spectral amplitudes for both beams, |*E*_1_(ω)| ≃ |*E*_2_(ω)|. The absolute phase spectra, without phase unwrapping, are reported in Figure 4c. The sawtooth shape of the phase of beam 1, ϕ_1_(ω), arises from the finite arrival time of the pulse at *t*_1_ = 2.3 ps, which introduces a linear phase term  in the frequency domain via the Fourier shift theorem.^20^

To compare the time-domain shapes of the pulses, and the absolute phase relative to the center of the THz pulse envelope, we calculated , using THz pulse arrival times *t*_*n*_ determined numerically as described in the Supporting Information and ref^20^. Performing the inverse DFT recovered the time-overlapped *E*_n_^*^(*t*), as shown in Figure 4d, where the similarity of the two pulses in the time domain is evident. The absolute phase spectra, ϕ_n_^*^(ω), are reported in Figure 4e, revealing that both pulses have similar frequency-dependent phases. At low frequencies, the near constant −π/2 phase is as expected for a single-cycle pulse,^20^ while the nonlinear increase in phase with frequency can be assigned to processes that introduce chirp, such as dispersion in the electro-optic crystal^21^ and the GaAs emitter.^18^ The two spectra can be compared by plotting the amplitude and phase of the ratio *R*(ω) = *E*_2_(ω)/*E*_1_(ω). The amplitude |*R*(ω)| is within 10% of the ideal value |*R*| = 1 over the range to 5.5 THz, with a small phase difference *∠R*(ω), as shown in Figure 4f.

The results above show that the spatial, temporal, and spectral properties of the two THz beams are very similar and that at higher frequencies (above 300 GHz), the two beams are spatially separated. Therefore, THz spectroscopy can be performed where one beam acts as a reference, while a sample is placed in the other beam.

### Dual-Beam Transmission and Baseline Correction

In standard single-beam THz-TDS, the transmission ratio *T*_single_(ω) = *E*(ω)/*E*_b_(ω) is obtained from spectra measured with a sample, *E*(ω), and without a sample, *E*_b_(ω). *T*_single_ is then inverted analytically or numerically to uncover the sample’s complex refractive index.^1,22^ As *E* and *E*_b_ are obtained sequentially, the assumption is made that the incident THz spectra have not changed in the intervening time between measurements, such that the true transmission of the sample is *T*(ω) = *T*_single_(ω). However, this is not the case if the amplitude or phase of the THz pulse produced by the spectrometer has altered in the time between acquiring the two spectra, such that the pulse incident on the sample, *E*_in_, is not the same as when measured previously, *E*_b_. Mathematically, *T*_single_ = *E*/*E*_b_ = *TE*_in_/*E*_b_ = η(ω)*T* where η(ω) = *E*_in_/*E*_b_ is the complex ratio of the fields incident on the sample during the two measurements. If η = 1, then the experimental transmission matches the actual transmission function of the sample, *T*_single_ = *T*, while η ≠ 1 implies that a systematic error has occurred in the time between measurements. As η is assumed to be unity in single-beam spectroscopy, this can lead to systematic errors in the optical properties of the sample deduced from *T*_single_ (shown later in the manuscript).

In dual-beam THz spectroscopy, we instead use four THz spectra to calculate the transmission
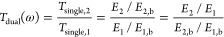
1where the baseline data *E*_1,b_ and *E*_2,b_ are acquired simultaneously for both beams without a sample and spectra *E*_1_ and *E*_2_ are acquired simultaneously when a sample is placed in beam 2. There are two ways to understand the dual-beam transmission based on the alternative ratios in eq 1. First, the ratio *E*_2_(ω)/*E*_1_(ω) corresponds to the transmission of the sample if both THz beams are identical in amplitude and phase. Dividing by the ratio *E*_2,b_/*E*_1,b_ corrects for the differences between the two beams. Alternatively, one can regard beam 1 as a reference beam that tracks variations in the amplitude or phase of the THz beam incident on the sample, with *T*_single,1_ = *E*_1_(ω)/*E*_1,b_(ω) = η_1_(ω) and hence
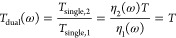
2where the last step was made assuming that η_2_(ω) = η_1_(ω). This is the case when the systematic error alters both THz beams in the same way, such as a variation in laser power or a change in atmospheric THz absorption caused by a variation in temperature, pressure, or humidity (since both THz beams traverse the same optical path). Having similar spectral performances for the two beams allows the reference beam to be used effectively over the same frequency range as the sample beam in dual-beam spectroscopy. Further, the extra phase of pulse 1 relative to pulse 2 in the raw data (Figure 4a) has no impact on *T*_dual_ as it contains the ratio *E*_1_/*E*_1,b_, which cancels out this extra phase.

### Robustness to Systematic Errors

**Figure 5:**
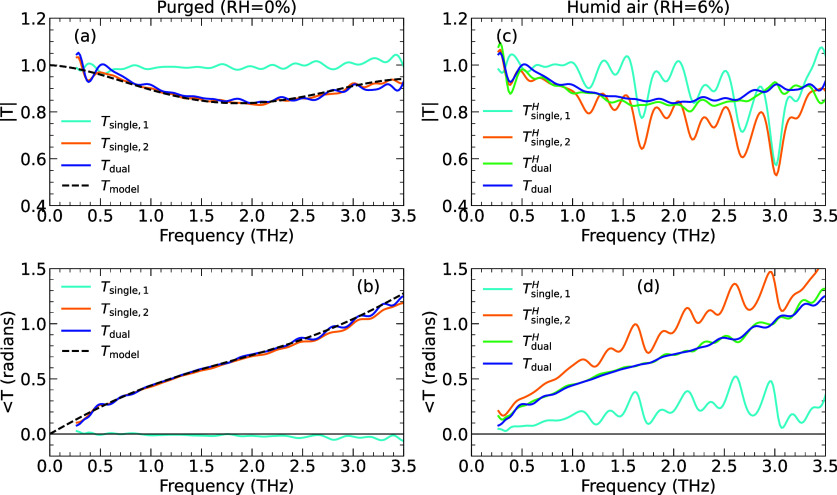
Relative performance of single-beam and dual-beam THz spectroscopy when measuring a thin plastic film, following a change in relative humidity (RH) that created a systematic error. (a) Amplitude transmission spectra for the plastic film when purged (RH = 0), showing the transmission for the reference beam (cyan, beam 1), single-beam transmission through the sample (orange, beam 2), and dual-beam transmission |*T*_dual_| (dark blue). The dashed black line shows the calculated transmission. (b) as (a) but showing the phase of the transmission. (c) Amplitude transmission spectra after changing the THz beam path to RH = 6%. Single-beam spectroscopy (*T*_single,2_^H^, orange) in the humid environment yields erroneous transmission minima near water vapor absorption lines. These were corrected using the simultaneous measurement of *T*_single,1_^H^ (cyan) to give dual-beam transmission *T*_dual_^H^, with an amplitude spectrum (green) in good agreement with the purged data from (a) (dark blue). (d) as (c) but showing the phase of the transmission.

To validate the practical performance of the dual-beam spectrometer approach, we report in Figure 5 the dual-beam spectroscopy of a 23 μm-thick sheet of the plastic polyethylene terephthalate (Mylar) in which changes were intentionally introduced to simulate the possible systematic errors that affect single-beam spectroscopy. We chose this plastic as it is readily available with standard thicknesses and produces only modest amplitude and phase changes: it is relatively thin and has low refractive index (low Fresnel reflection loss) and low absorption in the THz range. Amplitude and phase transmission spectra were first obtained with the THz beam path purged by dry nitrogen gas, as reported in Figure 5a,b. With the plastic sample placed in beam 2, |*T*_single,2_| (orange line) can be seen to reduce in amplitude to 0.86 at 2 THz, before increasing. This is not a consequence of any physical absorption in the sample: rather it is a consequence of the interference of multiple reflections within the plastic. The calculated transmission of the sample, made without assuming the thin-film limit,^1^ and assuming a frequency-independent complex refractive index *ñ* = 1.78 + 0.03i, is reported by the black dashed lines. This is in excellent agreement with the amplitude and phase of the single-beam sample transmission. The transmission of the reference beam, beam 1, has a modulus close to 1 and a phase close to zero (blue lines), indicating that no systematic error had occurred between the sample and baseline data acquisition. Hence, the dual-beam transmission *T*_dual_ (blue lines) is similar to *T*_single,2_ (orange lines).

Working under standard atmospheric conditions presents challenges for THz spectroscopy, imaging, and communications because of the rotational modes of water vapor.^23,24^ Variations in atmospheric temperature, pressure, or humidity cause the attenuation and dispersion of THz radiation to change at frequencies close to the numerous absorption lines in this frequency band. To explore the systematic error induced in single-beam THz spectroscopy as a result of varying humidity levels, we report in Figure 5c,d data acquired with 6% RH inside the THz spectrometer’s purge box. The amplitude and phase of the sequential, single-beam transmission *T*_single,2_^H^ can be seen to deviate from the true transmission, *T*_dual_ (obtained with full purge), at frequencies close to the water vapor absorption lines (1.7 THz, 2.2 THz, 2.7 THz, etc.). Here, the line widths were limited by the 20 ps scan window of the experiment. The systematic error has arisen because the sequential, single-beam spectroscopy measurement assumed the same humidity when the sample and baseline (reference) data were acquired, whereas in fact, *E*_2,b_ had RH = 0% and *E*_2_ was taken with RH = 6%. Even this small drift in RH lowers the relative THz amplitude to around 50% at 3.0 THz for this spectrometer’s 610 mm path length. This can be seen from *T*_single,1_^H^ (cyan) in Figure 5c and in more detail in the Supporting Information.

Considering now the dual-beam method: after the change in humidity, the dual-beam transmission *T*_dual_^*H*^ from eq 1 is shown by the green lines in Figure 5c,d and is very similar in modulus and phase to the “true” transmission of the sample as measured under purged conditions (dark blue). The effective correction for dual-beam spectroscopy was achieved because the measurement of the reference beam, *T*_single,1_^H^, contained the amplitude and phase changes created by extra water vapor in the spectrometer. Therefore, dual-beam spectroscopy can effectively eliminate systematic errors by using a reference beam that tracks a change in experimental conditions.

## Conclusions

We demonstrated an advanced dual-beam THz spectrometer that substantially reduced the systematic errors that can lead to reproducibility issues in standard single-beam THz time-domain spectroscopy. To evaluate the performance of our spectrometer, we intentionally introduced systematic errors in the amplitude and phase by altering the humidity level of the spectrometer, both of which produce significant systematic errors in conventional single-beam systems. Our dual-beam spectrometer was able to suppress these systematic errors by simultaneously measuring two THz beams that propagated along the same optical path, significantly enhancing the accuracy and reliability of the measurement data. Crucially, the low-aberration (*a*,*a*)(*a*,*a*) OAPM geometry achieved a diffraction-limited spot size, allowing independent sample and reference beams in the intermediate focal plane even with relatively small beam separations (3 mm here). Larger beam separations would be readily achievable by fabricating pixels spaced by a greater spacing or by cleaving devices and mounting them with known spacing. The beam profiles were studied using theoretical and experimental methods, in particular, identifying a small spatial overlap of the beams at lower frequencies. This challenge was circumvented experimentally via a modulation scheme that allowed the two beams to be individually resolved.

While this proof-of-concept was a free-space THz transmission setup based around a Ti:sapphire laser, our dual-beam approach can be readily extended to be compatible with fiber-coupled laser systems or to the reflection geometry. We recently demonstrated that fiber-coupled multipixel photoconductive antennas have comparable performance to commercial dipole antennas^14^ and can be used to generate different THz polarization states. A fiber-based beamsplitter would allow a two-pixel emitter in a compact package for use with Er:fiber lasers. On the optics side, while our OAPM geometry has good spatial resolution over a broad frequency range, the dual-beam concept is also applicable for lens-based THz optical systems, which are more straightforward to align but have lower signal-to-noise ratios at higher frequencies as a result of absorption in the lens material. The proposed dual-beam concept will find use in robust and reliable THz spectrometers for the spectroscopy and imaging of advanced materials.

## Data Availability

Data is available from the authors upon reasonable request.

## Supporting Information Available

THz beam propagation; frequency-dependent knife edge; Fourier transform theory; baseline correction; detection cross-talk; and robustness to systematic errors (PDF)

## References

[ref1] Lloyd-Hughes J.; Jeon T.-I. A Review of the Terahertz Conductivity of Bulk and Nano-Materials. J. Infrared, Millimeter, Terahertz Waves 2012, 33, 871–925. 10.1007/s10762-012-9905-y.

[ref2] Chen X.; Lindley-Hatcher H.; Stantchev R. I.; Wang J.; Li K.; Hernandez Serrano A.; Taylor Z. D.; Castro-Camus E.; Pickwell-MacPherson E. Terahertz (THz) biophotonics technology: Instrumentation, techniques, and biomedical applications. Chem. Phys. Rev. 2022, 3, 011311 10.1063/5.0068979.

[ref3] Withayachumnankul W.; Fischer B. M.; Lin H.; Abbott D. Uncertainty in terahertz time-domain spectroscopy measurement. J. Opt. Soc. Am. B 2008, 25, 1059–1072. 10.1364/josab.25.001059.

[ref4] Castro-Camus E.; Fu L.; Lloyd-Hughes J.; Tan H.; Jagadish C.; Johnston M. Photoconductive response correction for detectors of terahertz radiation. J. Appl. Phys. 2008, 104, 053113 10.1063/1.2969035.

[ref5] Mosley C. D. W.; Deveikis J.; Lloyd-Hughes J. Precise and accurate control of the ellipticity of THz radiation using a photoconductive pixel array. Appl. Phys. Lett. 2021, 119, 121105 10.1063/5.0064146.

[ref6] Jiang Z.; Li M.; Zhang X.-C. Dielectric constant measurement of thin films by differential time-domain spectroscopy. Appl. Phys. Lett. 2000, 76, 3221–3223. 10.1063/1.126587.

[ref7] Mickan S. P.; Lee K.-S.; Lu T.-M.; Munch J.; Abbott D.; Zhang X.-C. Double modulated differential THz-TDS for thin film dielectric characterization. Microelectron. J. 2002, 33, 1033–1042. 10.1016/S0026-2692(02)00108-8.

[ref8] Huang S.; Ashworth P. C.; Kan K. W. C.; Chen Y.; Wallace V. P.; Zhang Y.-T.; Pickwell-MacPherson E. Improved sample characterization in terahertz reflection imaging and spectroscopy. Opt. Express 2009, 17, 3848–3854. 10.1364/OE.17.003848. 19259226

[ref9] Chen X.; Pickwell-Macpherson E. An introduction to terahertz time-domain spectroscopic ellipsometry. APL Photonics 2022, 7 (7), 071101 10.1063/5.0094056.

[ref10] Chen H.; Wang K.; Chen X.; Fang G. Real-time and calibration-free generalized terahertz time-domain spectroscopic ellipsometry. Appl. Phys. Lett. 2024, 124, 111106 10.1063/5.0188364.

[ref11] Tu S.; Zhou J.; Rao X. Double-optical-path terahertz time-domain spectrometer. Chinese Patent CN 105548083 A2016,.

[ref12] Pengcheng N.; Chengyong C.; Hui Z.; Lei L.; Fangfang Q.; Tao D.; Xiaoxi L. System and method for detecting terahertz time-domain spectroscopy based on double light beams. Chinese Patent CN 109459406 A2019,.

[ref13] Mosley C. D. W.; Staniforth M.; Serrano A. I. H.; Pickwell-MacPherson E.; Lloyd-Hughes J. Scalable interdigitated photoconductive emitters for the electrical modulation of terahertz beams with arbitrary linear polarization. AIP Adv. 2019, 9, 45323 10.1063/1.5086428.

[ref14] Ou H.; Stantchev R. I.; Chen X.; Blu T.; Semtsiv M.; Masselink W. T.; Serrano A. H.; Costa G.; Young J.; Chopra N.; Lloyd-Hughes J.; MacPherson E. Simultaneous measurement of orthogonal terahertz fields via an emission multiplexing scheme. Opt. Express 2024, 32 (4), 5567–5581. 10.1364/OE.505567. 38439279

[ref15] Deveikis J.; Lloyd-Hughes J. Multi-pixel photoconductive emitters for the controllable generation of azimuthal and radial terahertz beams. Opt. Express 2022, 30 (24), 43293–43300. 10.1364/OE.473086. 36523030

[ref16] Chopra N.; Deveikis J.; Lloyd-Hughes J. Active THz beam shaping using a one-dimensional array of photoconductive emitters. Appl. Phys. Lett. 2023, 122, 061102 10.1063/5.0132207.

[ref17] Chopra N.; Lloyd-Hughes J. Optimum optical designs for diffraction-limited terahertz spectroscopy and imaging systems using off-axis parabolic mirrors. J. Infrared, Millimeter, Terahertz Waves 2023, 44, 981–997. 10.1007/s10762-023-00949-8.

[ref18] Shen Y. C.; Upadhya P. C.; Beere H. E.; Linfield E. H.; Davies A. G.; Gregory I. S.; Baker C.; Tribe W. R.; Evans M. J. Generation and detection of ultrabroadband terahertz radiation using photoconductive emitters and receivers. Appl. Phys. Lett. 2004, 85, 164–166. 10.1063/1.1768313.

[ref19] Brückner C.; Pradarutti B.; Müller R.; Riehemann S.; Notni G.; Tünnermann A. Design and evaluation of a THz time domain imaging system using standard optical design software. Appl. Opt. 2008, 47 (27), 4994–5006. 10.1364/AO.47.004994. 18806862

[ref20] Lloyd-Hughes J.; Chopra N. An accurate discrete Fourier transform to analyse the absolute phase of THz pulses. arXiv 2024, 2409.01950.

[ref21] Gallot G.; Zhang J. Q.; McGowan R. W.; Jeon T.-I.; Grischkowsky D. Measurements of the THz absorption and dispersion of ZnTe and their relevance to the electro-optic detection of THz radiation. Appl. Phys. Lett. 1999, 74, 3450–3452. 10.1063/1.124124.

[ref22] Withayachumnankul W.; Naftaly M. Fundamentals of measurement in terahertz time-domain spectroscopy. J. Infrared, Millimeter, Terahertz Waves 2014, 35, 610–637. 10.1007/s10762-013-0042-z.

[ref23] Xin X.; Altan H.; Saint A.; Matten D.; Alfano R. R. Terahertz absorption spectrum of para and ortho water vapors at different humidities at room temperature. J. Appl. Phys. 2006, 100, 94905 10.1063/1.2357412.

[ref24] Taleb F.; Alfaro-Gomez M.; Al-Dabbagh M. D.; Ornik J.; Viana J.; Jäckel A.; Mach C.; Helminiak J.; Kleine-Ostman T.; Kürner T.; Koch M.; Mittleman D. M.; Castro-Camus E. Propagation of THz radiation in air over a broad range of atmospheric temperature and humidity conditions. Sci. Rep. 2023, 13, 20782 10.1038/s41598-023-47586-8. 38012178 PMC10682482

