# Supplementary material for: Dual-Beam THz Spectrometer with Low-Aberration Optics and Off-Axis Multipixel Photoconductive Emitters for Reduced Systematic Errors
Source: ACS Photonics. 2025 Jan 11;12(2):917–24. doi: 10.1021/acsphotonics.4c01934 (PMC11843712; doi:10.1021/acsphotonics.4c01934)
Supplement: Supplementary file 1 — ph4c01934_si_001.pdf [file ph4c01934_si_001.pdf]

Supporting Information for:
A dual-beam THz spectrometer with low-aberration optics and off-axis multi-pixel photoconductive emitters for reduced systematic errors

Nishtha Chopra and James Lloyd-Hughes

6 pages, 5 figures.

I. THZ BEAM PROPAGATION

Gaussian beam propagation can be used to estimate the expected THz beam profile during propagation through a spectrometer. In the ABCD matrix approach, a set of matrices can be used to model a Gaussian beam as it diverges from a source in free space, or interacts with a lens [1]. To illustrate this approach we used the ABCD method to propagate a THz beam in a $4f$ setup, from an initial beam waist $w_0 = 0.4\,\text{mm}$ at the THz source, to a collimated beam path (using a 76.2 mm focal length perfect lens in place of an off-axis parabolic mirror), and on to a focus in the back focal plane of a second identical lens. The beam waist $w(z)$ (1/e radius of the electric field) is reported in Fig. S1 for different frequencies. Panel (a) shows the beam path from focus to focus, while panels (b) and (c) are zoomed near the focii, where the Gaussian beams reach their minimum values. As the same focal length was used for the two optics, the beam at the focus has unity magnification, with $\theta_2 = \theta_1$ and size $w_2 = w(z = 4f) = w_0$ at the second focus. A higher THz frequency (shorter wavelength) reduces the divergence of the initial beam, which is given by $\tan\theta_1 \simeq \theta_1 = \lambda/(\pi w_0)$, and hence the beam diameter in the collimated beam is smaller. The spot-size for Gaussian beams is then predicted to be independent of frequency, as higher frequencies do not fill the focusing optic (second lens/mirror) as effectively.

While this method gives some qualitative insights into the expected cross-sectional beam profiles at different positions, this method makes assumptions that are not valid in general for THz beam propagation:

1. The beam is assumed to always have a Gaussian profile while propagating.

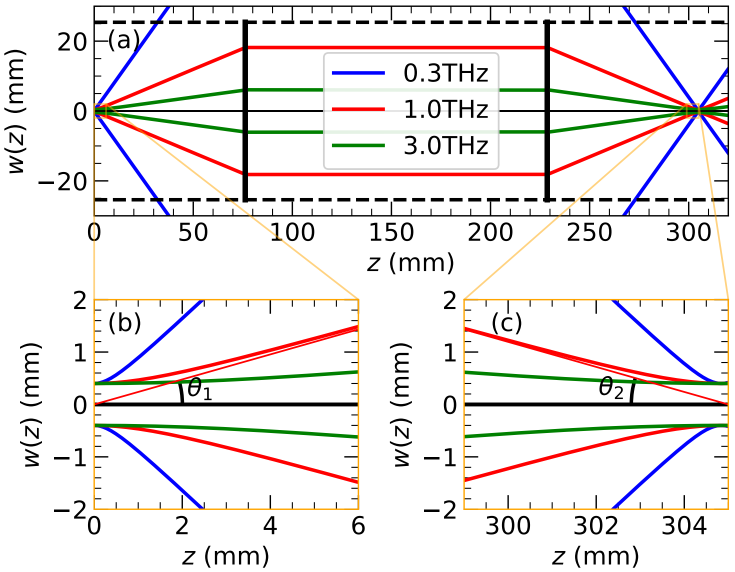

Fig. S1 (a) Gaussian beam propagating from an initial beam waist $w_0 = 0.4\,\text{mm}$ centred at $x = 0$, $y = 0$, travelling through a $4f$ system with $f = 76.2\,\text{mm}$, for different frequencies 0.3 THz (blue), 1.0 THz (red) and 3.0 THz (green). Dashed lines show the radius $R = 25.4\,\text{mm}$ of the optic, while solid black lines show lens/mirror positions. (b) Zoom near the THz source, showing the differing divergence angles for different frequencies. (c) Zoom near the focus, showing the focal beam waist w_2 is independent of frequency.

2. It assumes that the THz beam originates from an on-axis field point ($x = 0$, $y = 0$, $z = 0$), whereas off-axis sources are used in the dual-beam spectrometer presented here.

3. The divergence angle from the propagation direction (along z) has to be small, such that the paraxial approximation is valid. This is not the case at low frequencies (long wavelengths).

4. Real lenses and off-axis parabolic mirrors are assumed to be modelled by perfect, negligibly thin paraxial lenses.

5. The optics are assumed to have an infinite transverse extent (in x and y), rather than the finite size of real optics (e.g. radius R).

In experiments, the larger divergence of lower frequencies means that the beam profile propagating after the first optic cannot be Gaussian in shape (consider path at 0.3 THz in Fig. S1(a), where a large fraction of the beam extends past the radius $R = 25.4$ mm radius of the lens, and is hence lost). In the limit of low frequencies (e.g. 0.3 THz), the diffraction-limited spot size d is then given by $d \simeq \lambda / \sin\theta_2 = \lambda / \sin(R/\sqrt{(R^2+f^2)})$ for focal length f. Therefore the beam waist at the focus increases with wavelength, or scales inversely with frequency. At high frequencies, when the entire Gaussian beam is contained in the optical system, the smaller collimated beam diameter at higher frequency instead makes $w_2 = w_0$ (as stated above).

The limited validity of Gaussian beam theory motivated us to use optics simulations that can handle the propagation of non-Gaussian beams, as described in the main paper. However the Gaussian beam considerations presented here do allow the experimentally-observed frequency-dependence of the spot size (Fig. 3d) to be understood.

II. FREQUENCY-DEPENDENT KNIFE-EDGE

By acquiring THz time-domain scans of both beams as a knife-edge was translated horizontally along x, at a fixed position $z = 0$ in the sample plane, we obtained the frequency-dependent THz beam profiles. Time-domain electro-optic signals were Fourier transformed to obtain $E(f)$ at each x. Example spectra are shown in Fig. S2(a) for beam 2. By taking the amplitude at a fixed frequency as a function of the knife-edge position, we obtained the beam profiles shown in panels (b)-(d). As the signal corresponds to the integral of the THz electric field up to the knife edge position, the signal has the form of an error function (the integral of a gaussian). The black lines are error functions fit to the experimental data, and show standard deviations of $\sigma = 0.62 \pm 0.01$ mm at 0.3 THz, $\sigma = 0.24 \pm 0.01$ mm at 1.0 THz, and $\sigma = 0.15 \pm 0.01$ mm at 3.0 THz. While these values were averages for each beam, the data in Fig. 3(d) of the main text show the frequency-dependent values of σ_1 and σ_2 for each beam separately.

III. FOURIER TRANSFORM THEORY

In the main text we used an amplitude- and phase-accurate formulation of the discrete Fourier transform to calculate the Fourier spectra, and additionally corrected the arrival time of the two THz pulses to allow direct comparison of the two beams in the time-domain and frequency-domain (Figure 3). While these methods are presented in detail elsewhere [2], we describe them in brief here for completeness.

Discrete Fourier transform. The discrete time-series E_m contains the THz electric field sampled with n points spaced by time step δt, *i.e.* at times $t_m = m\delta t$ for $m = 0, 1, 2 ... n-1$. In the frequency domain the complex electric field $\widetilde{E}(\omega_k)$ sampled at discrete angular frequencies ω_k is

$$\widetilde{E}(\omega_k) = \delta t \cdot e^{-i\omega_k T_0} \sum_{m=0}^{n-1} E_m e^{i\omega_k t_m}, \tag{1}$$

where T_0 is the arrival time of the THz pulse relative to the start of the experimental time window. This definition differs from the standard definition of the discrete Fourier transform by the terms before the summation. The scaling by δt ensures that the amplitude spectrum is correct, while the phase factor $e^{-i\omega_k T_0}$ ensures that a single-cycle pulse arriving at time zero (*i.e.* an odd function) is purely imaginary and has a flat spectral phase of $\pm\pi/2$, as expected from the continuous Fourier transform [2]. The need for including information about the arrival time of the pulse can be understood by considering data sampled starting at different points (e.g. -20 ps or -10 ps): the standard FFT does not include knowledge of the absolute time of the THz pulse relative to the start of the time window, and hence different phases are obtained when the experimental time-window changes. This is not physically correct, as the absolute phase

of the THz pulse should be the same independent of the experimental sampling window. The exponential term makes this correction by introducing knowledge of the time window. The corresponding inverse discrete Fourier transform is

$$E_m = \frac{1}{n\delta t} \sum_{k=0}^{n-1} \widetilde{E}(\omega_k) e^{i\omega_k T_0} e^{-i\omega_k t_m}. \tag{2}$$

Application of the forwards then the inverse transform using Eqn. 1 then Eqn. 2 indeed recovers the time-domain pulse (shown in Fig. 2(a)).

Pulse arrival time. To find the pulse arrival time, T_0, relative to the first data point we calculated the first-order moment of the normalised intensity, $\langle t \rangle$, defined via:

$$T_0 = \langle t \rangle = \int_{-\infty}^{\infty} tI(t)dt = \frac{\int_{-\infty}^{\infty} tE^2(t)dt}{\int_{-\infty}^{\infty} E^2(t)dt} = \frac{\sum_{m=0}^{n-1} t_m E_m^2}{\sum_{m=0}^{n-1} E_m^2}. \tag{3}$$

Example data. In Fig. S3(a) the electric field of a typical time-domain pulse in beam 1 is reported (blue line) sampled with $n = 318$ points in steps of $\delta t = 25$ fs from -3.3 ps to 4.6 ps. After Fourier transform using Eqn. 1, the amplitude spectrum $|E(f)|$ is as shown in panel (b) (blue line, left y-axis), in units of the electric field. The red points (right-hand y-axis) show the amplitude calculated by the standard FFT routine, $|E_s(f)|$, using Eqn. 1 without the $\delta t \cdot e^{-i\omega_k T_0}$ term. The spectra have identical shape but have different scaling (by δt). The unwrapped spectral phase is reported in panel (c) over the range where the amplitude is above the noise floor (below 6 THz) [it is nonsensical to plot the phase if the amplitude hits the noise floor, where the phase is random]. The standard FFT incorrectly reports a large linear slope to the phase, $\arg[E_s(f)]$ (red points), because the standard FFT expression is relative to the first point in the time-domain series and hence has a linear slope given by $\arg[E_s(f)] = \omega T_0$ with $T_0 = 3.3$ ps (black line). In contrast, the spectral phase returned by the phase-accurate DFT of Eqn. 1 (blue) shows the absolute phase of the THz pulse, and is close to $-\pi/2$ since the pulse is close to an odd function and hence $E(f)$ is close to purely imaginary ($e^{-i\pi/2} = -i$). At higher frequencies the spectral phase increases as a result of chirp in the electro-optic sampling process in GaP, as well known in the literature and as further described in Ref. [2].

Time-shifting. The Fourier shift theorem states that for a function $E(t)$ with Fourier transform $\mathcal{F}[E(t)] = \widetilde{E}(\omega)$, if the function is shifted in the time-domain by $t \to t - T$, then the Fourier transform of the shifted function $E(t - T)$ can be written

$$\mathcal{F}[E(t-T)] = e^{i\omega T} \widetilde{E}(\omega). \tag{4}$$

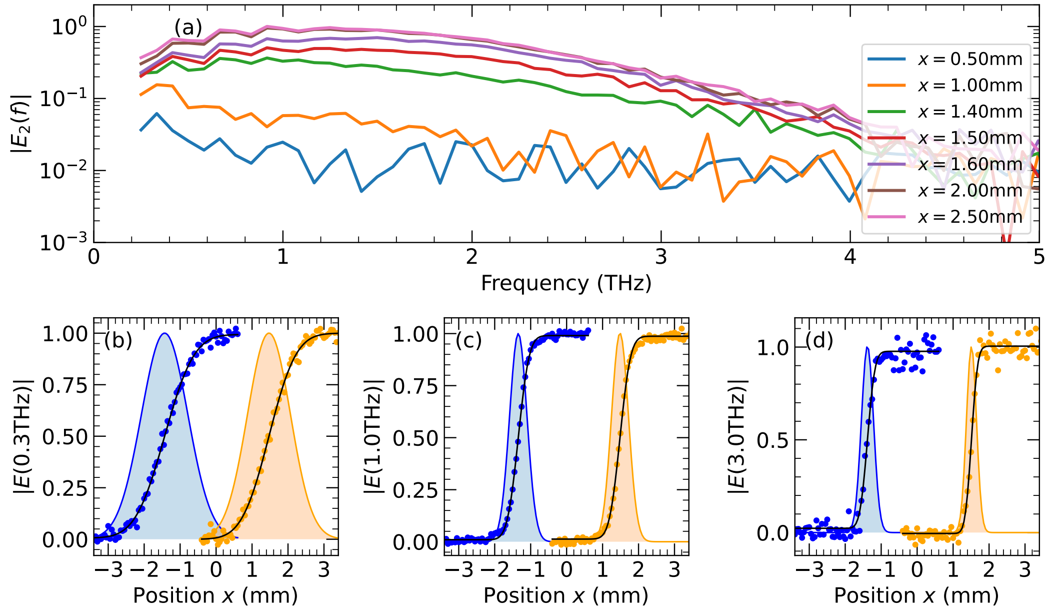

Fig. S2 (a) Amplitude spectra for beam 2 at different representative positions x of the knife edge. (b) Amplitude spectra at 0.3 THz for beam 1 (blue points) and beam 2 (orange points). The solid black line shows an error function fit, while the shaded gaussian areas show the derivative of the error function. (c) and (d) As (b), but at 1.0 THz and 3.0 THz.

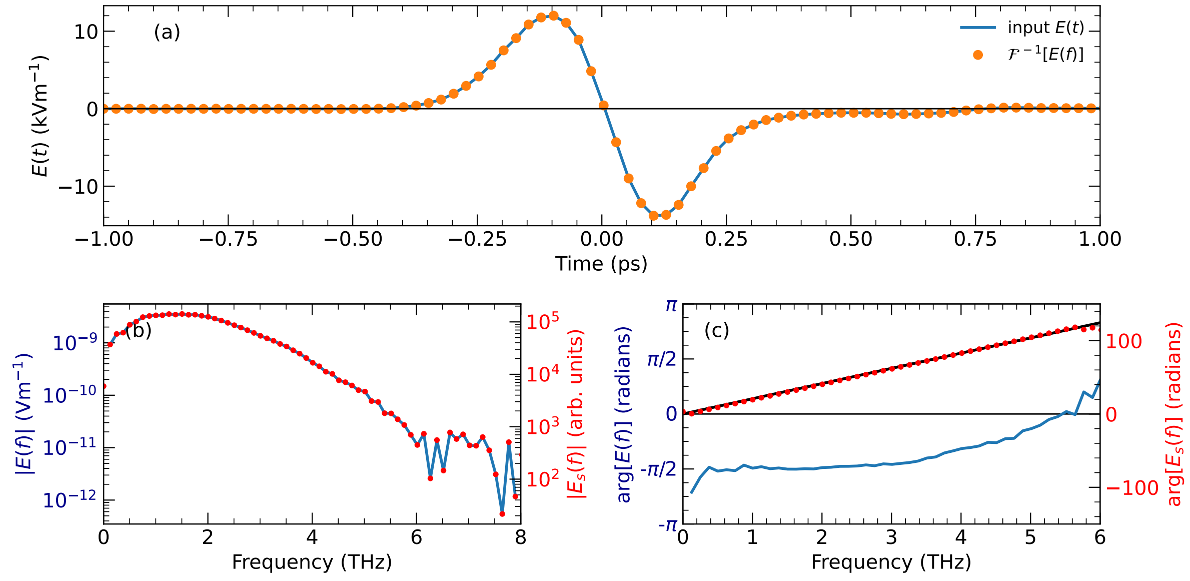

Fig. S3 (a) Typical time-domain data for beam 1, $E(t)$ (blue) and the recovered time-domain after forwards and inverse transform using Equations 1 and 2 (orange). (b) Spectral amplitude $|E(f)|$ in electric field units (Vm^{-1}) returned by Equation 1, and in arbitrary units as calculated by the standard FFT routine $|E_s(f)|$. (c) Spectral phase calculated by Equation 1 (blue line) and with the standard FFT routine (red points). The black line shows $\phi = \omega T_0$ with $T_0 = 3.3\,\mathrm{ps}$.

Thus the Fourier transform of the time-shifted function has had its linear phase changed by ωT relative to the unshifted Fourier transform. Conversely, multiplying by $e^{i\omega T}$ in the frequency domain shifts a THz pulse by time T in the time domain. This is used in the main text to allow the two THz pulses from beams 1 and 2, which arrived at different times, to be directly compared in the time-domain and in their spectral phase.

IV. BASELINE CORRECTION

Here we show that the complex transmission for the dual-beam spectrometer T_{dual}, as defined in the main text, can be written either using the unshifted spectra $E_n(\omega)$ or the time-shifted Fourier spectra, $E_n^*(\omega) = e^{-i\omega t_{n,b}} E_n(\omega)$, where $t_{n,b}$ is the arrival time for beams 1 and 2 in the baseline data. Starting from the definition of the dual-beam transmission using the time-shifted spectra, we find

$$T_{\mathrm{dual}}(\omega) = \frac{E_2^*}{E_1^*}\frac{E_{1,b}^*}{E_{2,b}^*} = \frac{e^{-i\omega t_{2,b}}E_2}{e^{-i\omega t_{1,b}}E_1}\frac{e^{-i\omega t_{1,b}}E_{1,b}}{e^{-i\omega t_{2,b}}E_{2,b}} = \frac{E_2}{E_1}\frac{E_{1,b}}{E_{2,b}} \tag{5}$$

and therefore the dual-beam transmission is the same whether the time-shifted spectra or unshifted spectra are used. Note that here we defined the time-shifted sample spectra as $E_2^* = e^{-i\omega t_{2,b}} E_2$ using the time shift of the baseline pulse, rather than as $E_2^* = e^{-i\omega t_2} E_2$, so that the extra phase accumulated through the sample (corresponding to the refractive index of the sample) is still present.

V. DETECTION CROSS-TALK

This can introduce cross-talk where a signal is measured when there should be none. As an example, Fig. S4 shows THz spectra acquired with both emitter pixels driven in-phase and demodulated at the same frequency (100 kHz), but with beam 2 (orange) blocked in the sample plane using a metal razor blade. In the top panel, cross-talk is evident at lower frequencies (orange line, below 2 THz) where the spectral amplitude was above the noise floor (dashed line). By using an orthogonal square wave voltage bias for pixel 2 compared to pixel 1, cross-talk was eliminated (lower panel, orange line) using the phase-sensitive nature of lock-in detection in quadrature.

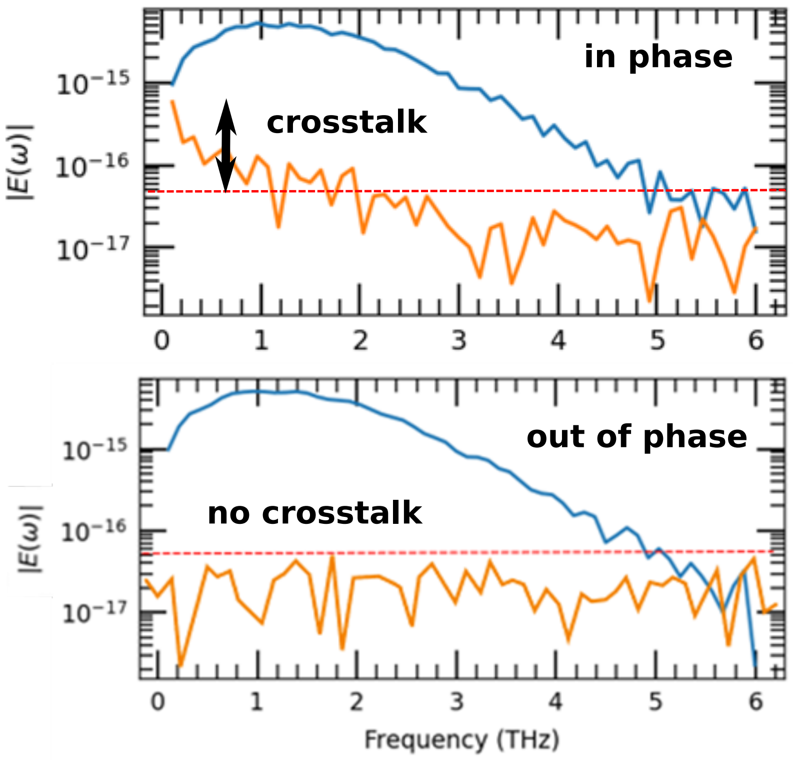

Fig. S4 THz spectra acquired via electro-optic sampling with THz emitter 1 and 2 on, probe beam 1 and 2 both on, but THz beam 2 physically blocked, for beam 1 (blue) and beam 2 (orange). Top panel: amplitude spectra when the emitter pixels are driven in-phase. The spectral amplitude at low frequencies arises from cross-talk in the detection crystal, where the tail of THz beam 1 produces an electro-optic signal in probe beam 2. Bottom panel: with emitter pixels driven in quadrature (out of phase) there is no discernible cross-talk.

VI. ROBUSTNESS TO SYSTEMATIC ERRORS

Firstly, a change in the amplitude of the emitted THz radiation was produced by lowering the voltage V applied to the photoconductive emitters.

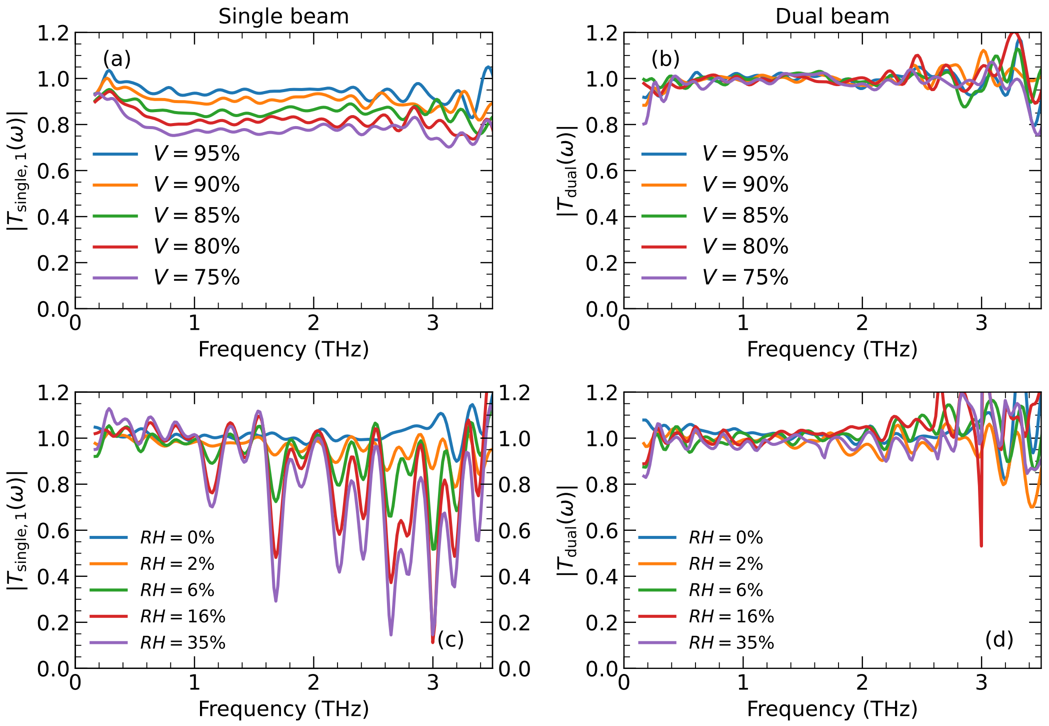

Fig. S5 Performance of single-beam and dual-beam THz setups when introducing systematic errors via amplitude or humidity changes. A transmission of 1 is desirable as it signifies no systematic error. (a) Amplitude of single-beam transmission using beam 1, $|T_{\mathrm{single},1}(V)| = |E_1(V)/E_{1,b}|$, for data taken when purged and at different voltages V as a percentage of the maximum voltage applied, creating amplitude variations (*e.g.* simulating changes in laser power). $E_{1,b}$ was obtained at maximum voltage. (b) Under the same conditions as (a), the dual-beam method obtained $|T_{\mathrm{dual}}| \simeq 1$, effectively removing the systematic amplitude error introduced. (c) $|T_{\mathrm{single},1}(RH)| = |E_1(RH)/E_{1,b}|$ shown on varying the atmospheric humidity from $RH = 0\,\%$ (fully purged) to $RH = 35\,\%$ (ambient humidity level). $E_{1,b}$ was obtained when fully purged. A variation in humidity produced a strong change in absorption at the water vapour lines, amounting to a systematic error in single-beam THz spectroscopy. (d) As (c), but for the dual-beam setup. $|T_{\mathrm{dual}}| \simeq 1$, showing that the dual-beam approach was less prone to systematic errors caused by humidity changes.

This simulated a reduction in the emitted THz strength, for example, as a result of lower fs laser power. Using just one beam, the ratio $T_{\mathrm{single},1} = E_1/E_{1,b}$ measured under nominally identical conditions defines the quality of the spectrometer's stability over the time frame of the measurements, with $T_{\mathrm{single},1} = 1$ expected if no systematic error has arisen since the acquisition of the baseline spectrum. Each spectrum was acquired from a 10 ps scan window in a data acquisition time of 10 s, providing sufficiently good signal-to-noise to analyse data from 0.2 THz to 3.5 THz. In Fig. S5(a) we report that $T_{\mathrm{single},1}(V) = E_1(V)/E_{1,b}$ lowered substantially when the emitter voltage was reduced (as the emission strength is proportional to the applied electric field). In a sequential single-beam THz spectroscopy, such amplitude variations could be misinterpreted, for instance, as extra absorption in the sample. In contrast, the dual-beam method records $T_{\mathrm{dual}}(V)$, where a variation in the amplitude or phase of beam 2 is corrected by measuring beam 1. As shown in Fig. S5(b), the dual-beam transmission is $|T_{\mathrm{dual}}| \simeq 1$, demonstrating the effectiveness of this method in eliminating systematic amplitude errors.

REFERENCES

[1] G. A. Brooker. *Modern Classical Optics.* Oxford Master Series in Physics. Oxford University Press, Great Clarendon Street, Oxford OX2 6DP, 2003.

[2] J. Lloyd-Hughes and N. Chopra. An accurate discrete Fourier transform to analyse the absolute phase of THz pulses. *arXiv*, 2024. `https://arxiv.org/abs/2409.01950`.
